# Sleep quality and athletic performance according to chronotype

**DOI:** 10.1186/s13102-020-00228-2

**Published:** 2021-01-07

**Authors:** Seung-Taek Lim, Do-Yoon Kim, Hyeong-Tae Kwon, Eunjae Lee

**Affiliations:** 1 Institute of Sport Science, Kangwon National University, Chuncheon, Gangwon-do Republic of Korea; 2 Waseda Institute for Sport Sciences, Waseda University, Tokorozawa, Saitama, Japan; 3 Nasaret International Hospital, Incheon, Republic of Korea; 4 Center for Sport Science in Incheon, 1F, Incheon Munhak Stadium, 618 Maesohol-ro, Michuhol-gu, Incheon, Republic of Korea 22234

**Keywords:** Sleep, Chronotype, Athletes, Elite, Performance

## Abstract

**Background:**

When studying the quality of sleep in relation to athletic performance, the athlete’s chronotype and habitual time consider important factors. We aim to investigate the sleep quality and athletes’ performance according to chronotype in elite athletes.

**Methods:**

Three hundred forty elite athletes (males = 261, females = 79) were recruited for the present study. All participants were screening for chronotype by the Korean versions of the Morningness - Eveningness Questionnaire (MEQ-K). The Pittsburgh Sleep Quality Index (PSQI) and Wingate Anaerobic Test (WAnT) were measurement after screening.

**Results:**

PSQI global score, PSQI sleep quality, PSQI sleep onset latency, PSQI sleep disturbance, and PSQI daytime dysfunction were significant differences among the groups. WAnT mean power (W), mean power (W/kg), peak power (W), and peak power (W/kg) were significant differences among the groups. A negative correlation coefficient was found between PSQI score and WAnT mean power (W) (*r* = − 0.256, *p* < 0.01), mean power (W/kg) (*r* = − 0.270, *p* < 0.01), peak power (W) (*r* = − 0.220, *p* < 0.01), and peak power (W/kg) (*r* = − 0.248, *p* < 0.01).

**Conclusions:**

This study indicates that related poor sleep quality and late-type chronotype may reduce the athletes’ performance in elite athletes. In addition, the sleep quality is much higher in the early-type chronotype than in the late-type chronotype. Moreover, it also the athletic performance was better in the early-type chronotype than in the late-type chronotype.

## Background

The amount and quality of sleep may affect performance, and there is a growing understanding of sleep patterns in elite athletes [1]. Sleep provides some important psychological and physiological functions that can be the basis of your recovery process [2]. In addition, a good night’s sleep is essential to control athletes’ hormones secretion and restoring metabolic processes in athletes [3].

Everyone has a biological or circadian rhythm, determined by various hormones [4]. Regulates the sleep/wake system and many other features such as blood pressure, hormone levels, body temperature, physical performance, alertness, mood, and intellectual ability to fluctuate during the day [5]. In general, humans have greater differences between individuals in timing of behaviors [6]. Previous studies reported that the effects of partial sleep deprivation (i.e., cognitive, physical, hormonal, and inflammatory responses to on various aspects of athletic performances) depend on time during the day, since evening performances decreased, but morning ones were unaffected [7, 8]. For athletes are very important these timing. Previous studies reported sixteen collegiate rowers had to perform a 2000-m rowing test, as result morning-type subjects rowed significantly faster than other type [9]. Henst et al. reported that endurance athletes who higher preference for the morning was related to the better individual best half marathon and the current marathon performance [10]. In additional, evening-type swimmers averaged 6% slower in the morning than evenings and had 50% higher α-amylase levels in the morning, morning-types required 5–7 times more effort in the evening test to achieve the same performance results as the morning test [11]. Humans are known to follow a circadian rhythm that peak performance occurs early in the evening and deteriorates in the afternoon [12]. When studying the quality of sleep in relation to athletic performance, the athlete’s chronotype and habitual time consider important factors [13].

All most athlete such as soccer, sprint, soccer, baseball, lacrosse, gymnastics, etc., can perform wingate anaerobic test (WAnT) and compare within athlete from “bad” to “excellent” for performance [14]. The standard 30-s WAnT’s 5-s and 30-s power output measurements are design to examine the first two of these energy reserves [15]. In typically, immediate results such as peak power (PP), which is the highest running in watts; mean power (MP) as the average power of the entire test in watts; power drop (PD) reduces power from beginning to end [16]. High post-exercise heart rate and high lactate concentration after the 30-s WAnT indicate superiority in glycosylation metabolism, thus indicating more value in the anaerobic capacity evaluation [17]. The system responsible for energy production is an anaerobic glycolysis and might be maintain for the rest of the total effort [18]. The WAnT is the most widely used test for evaluating the ability of human muscles to generate power in anaerobic energy systems [19], and it has been used in sports science research for over 30 years [20].

However, there are lacking published relate sleep quality and athletes’ performance with chronotype in elite athletes. Because of homogeneous patterns of chronotypes, difference in time to training each athletes, and so on which are many reasons. It has effects on the sleep quality and performance according to the athletes’ circadian rhythms, and is a very important consideration in the preparation of training schedules. Sleep quality is an individual’s subjective experience of sleep typically focusing on problem onset latency, sleep duration, efficiency, disturbance. Therefore, we classified the circadian rhythms using a chronotype questionnaire, and aimed to investigate the sleep quality and the athletes’ performance according to the chronotype classification. It tries to provide elite athletes with basic data on their training schedules and their own chronotypes. In this study, we hypothesized that sleep quality differs according to chronotypes and that there will be differences in athletes’ performance.

## Methods

### Participants

Three hundred forty elite athletes (males = 261, females = 79) were recruited for the present study. Athletes from basketball (*n* = 12), rugby (*n* = 22), wrestling (*n* = 5), boxing (*n* = 1), short track (*n* = 5), swimming (*n* = 14), squash (*n* = 8), baseball (*n* = 103), weight lifting (*n* = 3), judo (*n* = 6), soft tennis (*n* = 11), rowing (*n* = 34), canoe (*n* = 9), tennis (*n* = 1), fencing (*n* = 6), field hockey (*n* = 33), and handball (*n* = 67) were recruited. All participants were screened for chronotype using the Korean versions of the Morningness - Eveningness Questionnaire (MEQ-K) by Horne and Ostberg [21]: no definitely morning type (DM), thirteen moderately morning type (MM) (males = 11, females = 2), one hundred sixty-nine neither type (NT) (males = 136, females = 28), one hundred eleven moderately evening type (ME) (males = 75, females = 33), and sixty-one definitely evening type (DE) (males = 39, females = 16). There were no significant differences in age, height, weight, BMI and careers.

All subjects who agreed to participate in the study described the study to fully understand its purpose and the methods used in the ethical standards of the Declaration of Helsinki. In addition, all subjects signed an informed consent form prior to participation. This study was approved by Kangwon National University Review Board for Human Subjects (KWNUIRB-2020-03-007-002).

**Fig. 1:**
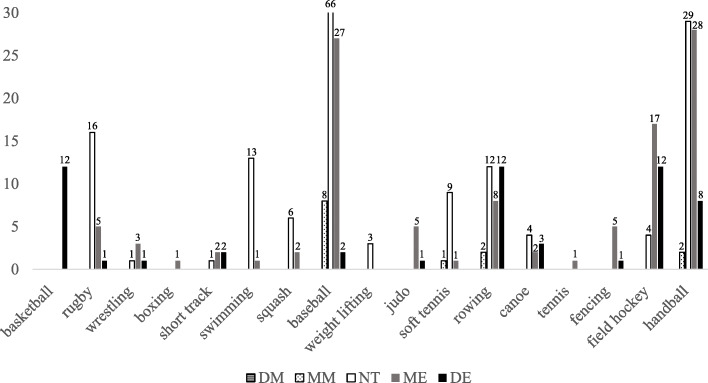
Frequency distribution of athletes’ chronotype according to sports

The characteristics of the participants are shown in Table 1, and frequency distribution of athletes’ chronotype according to sports are shown in Fig. 1.

**Table 1:** The characteristic of the participants

Variable	MM (*n* = 13)	NT (*n* = 164)	ME (*n* = 108)	DE (*n* = 55)	Total (*n* = 340)
Age (years)	15.92 ± 1.50	16.79 ± 3.43	18.03 ± 3.93	19.13 ± 4.30	17.55 ± 3.81
Height (cm)	174.5 ± 8.93	172.9 ± 7.63	172.6 ± 8.16	173.6 ± 8.24	173.0 ± 7.89
Weight (kg)	75.35 ± 13.0	73.67 ± 15.1	72.10 ± 14.1	73.46 ± 19.9	73.19 ± 15.0
BMI (kg/m^2^)	24.55 ± 2.50	24.49 ± 4.00	24.04 ± 3.48	24.09 ± 4.52	24.31 ± 3.80
Career (years)	6.46 ± 2.70	6.09 ± 3.40	6.76 ± 4.28	6.70 ± 4.71	6.42 ± 3.92

*MM* moderately morning type, *NT* neither type, *ME* Moderately evening type, *DE* definitely evening type, *BMI* body mass index

### Procedures

All participants conducted a measure of MEQ-K for screening chronotypes. In addition, PSQI and WAnT were measured. During the first visit to the laboratory, participants completed the informed consent form and measurement of MEQ-K for chronotypes. As a result of the chronotype, each participant’s time zone (DM for 0800 to 1000, MM for 0900 to 1100, NT for 1100 to 1300, ME for 1500 to 1700, and DM for 1700 to 1900) was designated for the best athletic performance and made a second visit. The time zone was set based on the MEQ-K questionnaire [21], participants asked to choose the time they could do the best performance. At the second visit, the height and weight for the WAnT test were measure, and the PSQI questionnaire was conduct. After that, each participant completed a self-selected stretching exercise and a five-minute cycle on the ergometer without applying a time limit for the WAnT. At the end of warm-up, all participants conducted WAnT experiment.

### Morningness - Eveningness questionnaire (MEQ)

The Korean Morningness - Eveningness Questionnaire (MEQ-K) from Horne and Ostberg used to assess the circadian typology of each subject [21]. MEQ has 19 items related to preferred time to participate in habitual physical and mental activities. MEQ scores range from 16 to 86, from extreme morning type to extreme evening type. The standard scores of the MEQ proposed by Horne and Ostberg were used to categorize the subjects as definitely morning type (DM; 70–86 score), moderately morning type (MM; 59–69 score), neither type (NT; 42–58 score), moderately evening type (ME; 31–41 score), and definitely evening type (DE; 16–30 score) [21]. Cronbach’s alpha for the MEQ-K was 0.77, and the correlation coefficient between the MEQ-K scores for verifying the test-retest reliability was 0.898.

### Pittsburgh sleep quality index questionnaire

The Pittsburgh Sleep Quality Index (PSQI) is a self-report questionnaire that evaluates sleep quality and quantity. The PSQI self-report questionnaire comprises of 19 items, yielding 7 component scores: (1) subjective sleep quality, (2) sleep latency, (3) duration, (4) habitual sleep efficiency, (5) sleep disturbances, (6) use of sleeping medication, and (7) daytime dysfunction. Each component is grade on a 0–3 severity scale based on the frequency of each disturbance and yields a global score with a range of 0–21 [22]. A PSQI global score of 5 or greater indicates a clinically significant sleep disorder who further screening is needed. Moreover, PSQI alone does not provide a reliable diagnosis [23].

### Wingate anaerobic test (WAnT)

WAnT was used following experiments performed by Kikuchi et al. [24]. The WAnT was performed on a cycle ergometer (Monark 824 E, Monark, Sweden) equipped a photoelectric sensor for recording 1.0 kg resistance basket and flywheel revolutions. Data for each 30-s WAnT were collected using POWER software (SMI, St Cloud, MN) and IBM-compatible microcomputer.

Each participant completed a self-selected stretching exercise and a five-minute cycle at the ergometer without applying a time limit. At the end of 1 min of warm-up, each participant performed an “all-out” sprint for 4 to 5 s to simulate the actual test.

Before starting of the WAnT, the resistance for each participant was calculate using a body weight of kilograms multiplied by male 7.5% and female 5%, and the determined amount was placed in the basket. At the start of the test, the assistant lifted the resistance basket and no resistance was applied to the flywheel, and each participant was instructed to begin pedal to reach the maximum rpm at the end of the 5 s countdown. The resistance basket was release, and data collection began, subsequently ending after 30 s. After 30 s WAnT, participants were instructed to pedal against light resistance (1.0 kg) until they returned to their pre-test condition.

### Statistical analysis

The SPSS statistical package version 25.0 for Windows (SPSS, Inc., Chicago, IL, USA) was used to perform all statistical evaluations. Means and standard deviations were computed for all variables, and normality was checked with the Shapiro Wilk test. Non-normal data were converted using square root or logarithmic transformations which achieved normality for all variables. Sleep state and wingate anaerobic power by chronotype were verified through a one-way analysis of variance (ANOVA). The relationships among variables were analyzed using Pearson’s correlation coefficients. Post-hoc analysis (Bonferroni test) was used to compare specific differences when significance was found. Statistical significance was accepted at the 0.05 level.

## Results

### Sleep state according to chronotype

The sleep state according to chronotype is present in Table 2. One-way ANOVA showed that PSQI global score (*p* < 0.001), PSQI sleep quality (*p* < 0.001), PSQI sleep onset latency (*p* < 0.001), PSQI sleep disturbance (*p* = 0.002), and PSQI daytime dysfunction (*p* = 0.005) were significantly difference in among the groups. However, no significant difference in PSQI sleep duration, PSQI sleep efficiency, and PSQI use of medications. Post-hoc analysis using Bonferroni test indicated that PSQI global score, PSQI sleep quality, and PSQI sleep onset latency in MM group were significantly lower than DE group.

**Table 2:** Sleep state according to chronotype

Variable	Groups				
	MM (*n* = 13)	NT (*n* = 164)	ME (*n* = 108)	DE (*n* = 55)	*p*-value
PSQI global score	2.46 ± 2.26^a^	3.14 ± 2.00^c,d^	4.09 ± 2.15	5.05 ± 3.23	< 0.001
PSQI sleep quality	0.62 ± 0.51^a^	0.83 ± 0.59^c,d^	1.06 ± 0.58	1.21 ± 0.61	< 0.001
PSQI sleep onset latency	0.15 ± 0.38^a,b^	0.62 ± 0.71^c,d^	0.97 ± 0.87	1.16 ± 1.08	< 0.001
PSQI sleep duration	0.23 ± 0.83	0.30 ± 0.70	0.41 ± 0.76	0.48 ± 0.94	0.376
PSQI sleep efficiency	0.31 ± 0.85	0.16 ± 0.57	0.16 ± 0.48	0.34 ± 0.87	0.187
PSQI sleep disturbance	0.77 ± 0.44	0.85 ± 0.57^d^	1.01 ± 0.50	1.13 ± 0.62	0.002
PSQI use of medications	0.15 ± 0.55	0.05 ± 0.33	0.02 ± 0.13	0.08 ± 0.38	0.337
PSQI daytime dysfunction	0.23 ± 0.60	0.33 ± 0.55^d^	0.46 ± 0.66	0.64 ± 0.68	0.005

*PSQI* Pittsburgh Sleep Quality Index, *MM* moderately morning type, *NT* neither type, *ME* Moderately evening type, *DE* definitely evening type, *BMI* body mass index

^a^MM vs DE; ^b^MM vs ME; ^c^NT vs ME; ^d^NT vs DE

### Wingate anaerobic power according to chronotype

The wingate anaerobic power according to chronotype is present in Table 3. One-way ANOVA showed that power drop (%) (*p* < 0.001), mean power (W) (*p* < 0.001), mean power (W/kg) (*p* < 0.001), peak power (W) (*p* < 0.001) and peak power (W/kg) (*p* < 0.001) were significantly difference in among the groups. Post-hoc analysis using Bonferroni test indicated that power drop (%), mean power (W), mean power (W/kg), and peak power (W/kg) in MM group were significantly higher than DE group.

**Table 3:** Wingate anaerobic power according to chronotype

Variable	Groups				
	MM (*n* = 13)	NT (*n* = 164)	ME (*n* = 108)	DE (*n* = 55)	*p*-value
Power drop (%)	30.42 ± 11.22^a^	31.71 ± 9.02^d^	32.89 ± 8.62^e^	37.71 ± 5.12	< 0.001
Mean power (W)	570.7 ± 153.7^a^	526.3 ± 136.8^c,d^	477.3 ± 135.0	454.8 ± 103.0	< 0.001
Mean power (W/kg)	7.30 ± 1.38 ^a^	6.91 ± 1.12^c,d^	6.47 ± 1.13	6.10 ± 0.75	< 0.001
Peak power (W)	703.2 ± 177.0	655.6 ± 171.1	612.2 ± 158.5	603.5 ± 151.4	< 0.001
Peak power (W/kg)	9.04 ± 1.75 ^a^	8.76 ± 1.23^c,d^	8.28 ± 1.25	8.03 ± 1.04	< 0.001

*MM* moderately morning type, *NT* neither type, *ME* Moderately evening type, *DE* definitely evening type, *BMI* body mass index

^a^MM vs DE; ^b^MM vs ME; ^c^NT vs ME; ^d^NT vs DE; ^e^ME vs DE

### Correlations coefficients between the PSQI score and Wingate anaerobic power

Table 4 shown that the correlation coefficients of the PSQI score and wingate anaerobic power. A negative correlation coefficients was found between PSQI score and mean power (W), mean power (W/kg), peak power (W), and peak power (W/kg) (*p* < 0.01; *p* < 0.01; *p* < 0.01; *p* < 0.01, respectively).

**Table 4:** Correlations coefficients between the PSQI score and wingate anaerobic power

Variable	PSQI	MP (W)	MP (W/kg)	PP (W)	PP (W/kg)
PSQI	–				
MP (W)	−0.256^**^	–			
MP (W/kg)	−0.270^**^	0.804^**^	–		
PP (W)	−0.220^**^	0.949^**^	0.693^**^	–	
PP (W/kg)	−0.248^**^	0.770^**^	0.894^**^	0.791^**^	–

*PSQI* Pittsburgh Sleep Quality Index, *MP* mean power, *PP* peak power

**: *p* < .01

## Discussion

In the current study, we examined the sleep quality and athletes performance according to chronotype in elite athletes. The main finding of the study that PSQI global score, PSQI sleep quality, PSQI sleep onset latency, PSQI sleep disturbance, and PSQI daytime dysfunction were significantly difference in chronotype. Also, WAnT various (power drop, mean power and peak power) were definitely significantly difference in chronotype. In addition, a negative correlation found between PSQI score and WAnT.

Sleep is an important factor in improving athletic performance. Human ability to cope with physiological and psychological stressors is important for the results of athletic performance [25]. It is affect by several factors, including natural fluctuations in physiological and behavioral processes (for example, sleep-wake cycle, body temperature, hormonal regulation) for 24 h’ period [26]. As a result, poor sleep quality and delay circadian sleep phase of athletes from adolescence to adulthood, suggests that the decreased athletic performance substantial [27]. In this study found that PSQI global score, sleep quality, and sleep onset latency were better in MM more than late other type (NT, ME, and DE) (Table 2). Moreover, a negative correlation between PSQI score and WAnT (Table 4). Previous studies, the prolonged period of sleep deprivation is associated with increased sympathetic, decreased parasympathetic cardiovascular control, and spontaneous discomfort sensitivity in healthy adults [28]. Oda and Shirakawa reported that a delayed onset of sleep, significant physiological excitement of sleep time due to increased heart rate, results indicate that they may causes a large physiological excitement during sleep time and interfere with the onset of sleep [29]. This further supports the findings of Hausswirth et al. the decrease in sleep time can be cause by a decrease in efficiency, mainly due to the difficulty of staying stationary during sleep [30]. In additional, improved specific measures of basketball performance after extended sleep may help optimal sleep to reach peak athletic performance [31]. We observed negative correlation between poor sleep quality and athletic performance. The PSQI questionnaire used in the sleep quality test is widely used by athletes. PSQI questionnaire is easy to use in the field and are shown largely in the verification of the difference (effect size, 0.36) between athletes and non-athletes, and are useful for examining the quality of sleep for athletes [32].

However, PSQI questionnaires are subjective, so it is difficult to know the exact sleep quality. It is necessary to examine the sleep quality through objective and scientific methods such as polysomnography (PSG), activity monitoring, and consumer sleep technology [33]. The standard of the quality evaluation of sleep is known as the PSG. However, full PSG is difficult to use as a measurement method is difficult and expensive equipment, but it is widely used in sports fields due to the development of a portable and easy to measure PSG device [34]. Moreover, the specificity of training and competition schedules differs from each athlete, it should be evaluated through an activity monitors (actigraphy) [35].

Chronotype is an individual difference that reflects the time that an individual is “does his or her best” [36]. Reilly and Waterhouse describe that performance changes are simultaneously affected by other multi-factorial systems, such as external (exogenous), internal (endogenous), and psychobiological (lifestyle) mechanisms [37]. We classified athletes according to chronotype by MEQ-K, based on the fact that athletes’ performance is affecte by individual time differences. Found that power drop (%), mean power (W), mean power (W/kg), peak power (W) and peak power (W/kg) were significantly highest in MM more than late other type (NT, ME, and DE) (Table 3). Cortisol, considered an indicator of psychophysiological stress and associated with poor sports performance, shows the peak of early morning under normal conditions [38]. Also, suggested that evening-types more time needed to prepare for physical activities or training after waking up rather than in the morning [39]. In other reason that evening-type may have a shorter sleep time during daily activity than other chronotypes [40]. This may further delay the circadian rhythm since the evening-type is reluctant to advance bedtime [41]. Results from previous studies and this study showed significant differences in athletes’ performance according to the chronotype. The significant difference between the morning type and the evening type of chronotype is sleep onset latency (Table 2). Alexandru et al. reported that strong relationship between sleep-wake patterns dysfunctions and sleep onset latency increase [42]. Eventually, an increase in sleep onset latency adversely affects sleep quality.

Training and competition schedules may affect athletes differently. It is participating in a sport that matches athletes’ chronotype is more likely to exhibit optimal performance than an athlete who participates in a sport that is opposite to his or her chronotype [6]. An athlete’ chronotype could enhance the competitive, understanding their own individual chronotype tendencies might allow athletes to arrange training schedules. In this study, the morning type showed higher sleep quality than the evening type, so the evening type athletes are recommended to improve their sleep quality and change to morning type life.

The present study has some limitations and points to suggestions for further research. We did not control such factors as their normal lifestyle, training schedule, and smoking. We assumed that because the subject has normally rhythm for athletes. Moreover, we also recommend that all participants avoid drastic changes in their lifestyle for about 1–2 weeks prior to the questionnaire. Another limitation is that did not distinguish between male and female. Further research on gender is needed. Moreover, chronotype is classified into 5 types. However, there was no definitely morning type among the participants in this study. In future studies, it is necessary to investigate the relationship between sleep quality and performance in all types of chronotype.

## Conclusion

In conclusion, this study indicates that related poor sleep quality and late-type of chronotype may reduce the athletes’ performance in elite athletes. In addition, the sleep quality is much better in the early-type chronotype than in the late-type chronotype. Moreover, it also the athletic performance was better in the early-type chronotype than in the late-type chronotype. Athletes know their chronotype and, if possible arrange a training schedule at that time will be effective in improving performance. To this end, athletes must be encouraged to have a regular lifestyle, especially at the time when their sports events opening mainly. Moreover, in future studies, it is necessary to find out how the intervention studies affect athletes’ performance and sleep quality according to chronotypes.

## Data Availability

Full data for this research is available through the corresponding author upon request.

## Abbreviations

ANOVA: Analysis of variance
BMI: Body mass index
DE: Definitely evening type
DM: Definitely morning type
MEQ-K: Korean versions of the morningness - eveningness questionnaire
MP: Mean power
ME: Moderately evening type
MM: Moderately morning type
MEQ: Morningness - eveningness questionnaire
NT: Neither type
PP: Peak power
PD: Power drop
SPSS: Statistical package for the social sciences
PSQI: The pittsburgh sleep quality index
WAnT: Wingate anaerobic test
